# The Importance of SNPs at miRNA Binding Sites as Biomarkers of Gastric and Colorectal Cancers: A Systematic Review

**DOI:** 10.3390/jpm12030456

**Published:** 2022-03-14

**Authors:** Fatemeh Hajibabaie, Navid Abedpoor, Nazanin Assareh, Mohammad Amin Tabatabaiefar, Laleh Shariati, Ali Zarrabi

**Affiliations:** 1 Department of Physiology, Medicinal Plants Research Center, Isfahan (Khorasgan) Branch, Islamic Azad University, Isfahan 81595-158, Iran; fateme.hajibabaii1991@gmail.com (F.H.); nazaninassareh@gmail.com (N.A.); 2 Department of Sports Physiology, Faculty of Sports Sciences, Isfahan (Khorasgan) Branch, Islamic Azad University, Isfahan 81595-158, Iran; abedpoor.navid@gmail.com; 3 Department of Genetics and Molecular Biology, School of Medicine, Isfahan University of Medical Sciences, Isfahan 81746-73461, Iran; mamintab@yahoo.co.uk; 4 Pediatric Inherited Diseases Research Center, Isfahan University of Medical Sciences, Isfahan 81746-73461, Iran; 5 Department of Biomaterials, Nanotechnology and Tissue Engineering, School of Advanced Technologies in Medicine, Isfahan University of Medical Sciences, Isfahan 81746-73461, Iran; 6 Biosensor Research Center, School of Advanced Technologies in Medicine, Isfahan University of Medical Sciences, Isfahan 81746-73461, Iran; 7 Biomedical Engineering Department, Faculty of Engineering and Natural Sciences, Istinye University, Sariye, Istanbul 34396, Turkey

**Keywords:** colorectal cancer, gastric cancer, miRNA, single nucleotide polymorphism, biomarkers

## Abstract

Dysregulated mRNA–miRNA profiles might have the prospective to be used for early diagnosis of gastrointestinal cancers, estimating survival, and predicting response to treatment. Here, a novel biomarker based on miRNAs binding to mRNAs in single nucleotide polymorphism (SNP) sites related to gastrointestinal cancers is introduced that could act as an early diagnosis. The electronic databases used for the recruiting published articles included EMBASE, SCOPUS, Web of Science, and PubMed, based on MESH keywords and PRISMA methodology. Based on the considered criteria, different experimental articles were reviewed, during which 15 studies with the desired criteria were collected. Accordingly, novel biomarkers in prediction, early prognosis, and diagnosis of gastrointestinal cancers were highlighted. Moreover, it was found that 20 SNP sites and 16 miRNAs were involved in gastrointestinal cancers, with altered expression patterns associated with clinicopathological and demographic data. The results of this systematic study revealed that SNPs could affect the binding of miRNAs in the SNP sites that might play a principal role in the progression, invasion, and susceptibility of gastrointestinal cancers. In addition, it was found that the profiles of SNPs and miRNAs could serve as a convenient approach for the prognosis and diagnosis of gastric and colorectal cancers.

## 1. Introduction

According to the World Health Organization statements, cancers rank second in non-communicable diseases (NCD) after cardiovascular disorder [1]. Cancers are characterized by uncontrolled cell division and abnormal growth with non-clear inheritance patterns [2]. Although family history is defined as the principal genetic characteristic in the etiology of cancer inheritance [3], some environmental risk factors, including a Western diet, smoking habits, sedentary lifestyle, and alcohol consumption, could negatively influence genetics and epigenetics expression patterns [4,5,6]. Therefore, the mentioned risk agents have been identified as carcinogenic factors. Gastrointestinal neoplasms are heterogeneous tumors in the digestive tract organs with multi-grade and multi-stage processes established under biological and environmental carcinogenic and mutagenic factors [7].

Colorectal cancer is the most prevalent cancer of digestive system with multifactorial etiology and complex pathological indexes. Different diagnoses and therapeutic approaches are used for this type of cancer, which are based on genetic factors, familial history, and environmental terms, including gut microbiota alteration, diet pattern, infection, metabolic disorders, and inflammation [6]. Global cancer statistics data (GLOBOCAN) mentioned that colorectal cancer is estimated as the third most common cancer in the world with a continues rise in developing countries [8]. It is also known as the second most deadly cancer, with most deaths in Eastern Asia [5]. Stomach cancer, termed as gastric cancer, is the fifth-highest prevalent cancer and is classified as the third cause of mortality following colorectal cancer [8].

Furthermore, it has the highest incidence rate in East and Central Asia and Latin America [8,9]. New data have indicated that more than 50 loci with genetic variability in the human genome are related to gastrointestinal carcinoma susceptibility. [10]. Moreover, microarrays and high-throughput data analysis have highlighted coding and non-coding RNAs with differential expressions in neoplasm pathogenesis. Several studies have revealed that miRNAs are involved in crucial pathways and comprehensive biological processes, including apoptosis, reprogramming gene expression, differentiation, proliferation, cell cycle, tumorigenesis, diseases development, cancer pathogenesis, and aging [11,12,13,14]. MiRNAs are a class of non-coding RNAs with about 20 to 25 nucleotides involved in modulating biological processes through alteration in post-translational regulation. [15]. MiRNAs bind primarily to the 3’ untranslated region (3’ UTR) of the genes through seed sequences and regulating gene expression [16,17]. Variation in the single-nucleotide position of the DNA sequence with multi-allelic genotypes among the general population is termed as single nucleotide polymorphism (SNP). SNPs are located in the coding and non-coding regions of genomes. The existence of SNP in target regions of miRNAs could result in the regulation and alteration of gene expression that are the critical points in the pathogenicity of diseases [18]. Based on solid evidence, the occurrence of single-nucleotide variation in miRNA binding sites via alteration in the binding affinity to SNP sites and post-transcriptional dysregulations could affect carcinogenesis risk, survival score, and cancer invasion [19]. In this systematic review, we focused on the influence of SNPs in the binding site of miRNAs and the role of miRNAs binding to SNP sites on gastrointestinal cancer susceptibility.

## 2. Materials and Methods

**Figure 1:**
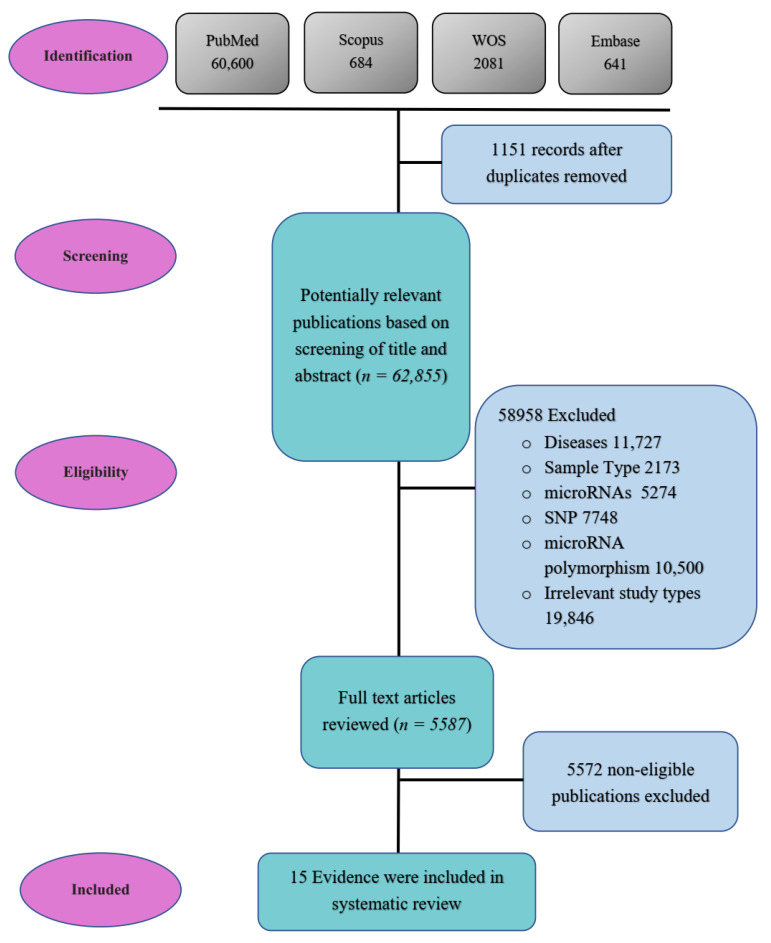
The flowchart of the systematic review. We indicated the algorithm for identifying relevant papers for inclusion. Based on the comprehensive literature, 15 eligible pieces of evidence were included for the effect of the SNPs in the binding site of miRNAs and the role of miRNAs binding to SNP sites on gastrointestinal cancer susceptibility.

This systematic review was conducted based on the preferred reporting items for systematic reviews (PRISMA) and guidelines on systematic reviewing methodology. A flowchart was designed to report the procedure and identify the literature strategy (Figure 1). 

### 2.1. Search Strategy

In this study, we tended to evaluate MiRNA-related polymorphisms in gastrointestinal cancer; thereby, four electronic databases, including EMBASE, SCOPUS, WEB OF SCIENCE, and PubMed, were considered for articles published between 2010–2021. In this systematic review, the search strategy used for colorectal cancer and gastric cancer was following medical subject headings (MeSH), including the input terms: colorectal cancer, gastric cancer, single nucleotide polymorphism, and miRNA, which were performed in databases such as PubMed, Scopus, Web Of Science, and subsequently Embase. 

### 2.2. Study Selection

#### 2.2.1. Inclusion Criteria

We selected case-control studies that evaluated the expression of miRNA and genes and the effect of SNP in the miRNAs binding to the SNP sites. In addition, studies that measured the relative expression of genes and miRNAs bound to SNP sites in gastrointestinal cancers were also included. Moreover, articles in English published between 2010–2021 were selected. 

#### 2.2.2. Exclusion Criteria

This systematic review selected research based on the main criteria, the potentially functional miRNA binding to polymorphism sites, and removed other studies without these criteria. We excluded studies that predicted the miRNAs or SNPs and did not measure the expression level of miRNAs. In addition, the studies that measured miRNAs without SNP genotyping methods and presented polymorphisms in miRNA sequences were excluded. Interpretation studies, review articles, and case reports after screening their related articles and citation lists for relevant original investigations were excluded. In addition, preclinical and in vitro studies were removed.

#### 2.2.3. Data Extraction

After the screening of the studies, the relative variables were extracted by two independent investigators and standardized electronic format independently. The relative variables included the first author’s name, publication year, region, design, population (sex, age, race, and sample size), exposure assessment, exposure categories, demographic characteristics of participants, outcome definition, outcome ascertainment, and statistical analysis method. We also detected the SNPs in the seed region of miRNA binding sites on their targeted genes for each study.

### 2.3. Quality Assessment

Two independent reviewers duplicate-screened the title and abstract in this systematic review. Then, we assessed the evidence based on the full text and selected studies according to the eligibility criteria. First, we evaluated the quality of the study based on four eligibility criteria, including evaluation of outcome, evidence of bias, validation of outcome, and appropriateness and reporting of exclusion and inclusion as previously reported (10). The quality score was described as 0–5, in which quality scores from 0 to 3 were considered low quality, and 4 to 5 were generally considered high quality. Moreover, we assessed the risk of individual bias studies by using the Newcastle–Ottawa Scale (N.O.S.). We categorized the N.O.S. into three stages: (1) studies with 7–9 stars were considered low risk of bias, (2) evidence with 5–6 stars were deemed moderate risk of bias, and (3) studies with 0–4 stars were considered high risk of bias.

## 3. Results

This systematic research considered studies that evaluated SNP frequency by genotyping methods. Moreover, we included studies that assessed miRNAs that have a binding affinity to 3’ UTR of targeted genes and measured the expression of these targeted genes. Hence, we found 64,006 studies about polymorphisms in the miRNA binding site of susceptible genes involved in colorectal and gastric cancers at the first stage. After applying our inclusion and exclusion criteria, in the final screening stage, we collected 15 studies with the desired criteria.

### 3.1. Colorectal Cancer

The rs141178472 is located at the 3’ UTR of the PIK3CA gene and is targeted by miR-520a. Considering the role of the PIK3CA gene and miRNAs, variation in 3’ UTR of this gene could increase susceptibility to colorectal cancer in the Chinese population. Lifang Ding et al. have indicated that the genotype frequency of rs141178472 between 386 cases with colorectal cancer and 394 control cases in the China population has corresponded to Hardy–Weinberg equilibrium [20]. Based on the results of this study, the T allele in this polymorphism was dominant. Genotypic results of this study revealed no significant association between rs141178472 genotypes and the tumor stage, while cases with CC genotype in rs141178472 were significantly exposed with a high risk of colorectal cancer in comparison to the carriers with wild-type homozygous alleles (T.T.). Furthermore, individuals with heterozygous C allele also were revealed to have higher risk of colorectal cancer (C.R.C.) than the heterozygote T allele. Thus, allele T in rs141178472 could make a new binding site for miR-520a and decrease the expression of PIK3CA. Moreover, the relative expression of miR-520a was verified by the Luciferase reporter gene plasmids constructed method and real-time qPCR into 293T cells. 

Conversely, in the cohort study, including 831 patients, the single-nucleotide variation rs12373 in the 3′ UTR region of the PAUF gene made a new binding site for miR-571. This could modulate the PAUF gene expression and might be a possible prognostic biomarker for colorectal cancer patients [21]. Accordingly, the recognized variation A > C in rs12373 in the 3′ UTR region of the PAUF gene was significantly related to prognosis outcome in the discovery cohort. To confirm whether SNP rs12373A > C enhanced binding efficiency of miR-571 on 3′ UTR of PAUF gene, a Renilla-Luciferase assay was conducted, and outcomes were revealed that the activity of the Renilla reporter was significantly higher in the A.A. allele compared to the CC construct. The PAUF gene is a novel oncogenic secretion in many cancers, especially pancreatic cancer. Experimental research demonstrated that this oncogene is overexpressed in malignant tumors and leads to the metastatic process. In addition, Jong Gwang Kim et al. showed that according to the multivariate survival statistics in all patients such as pathological stage, carcinoembryonic antigen (C.E.A.) marker levels, and age, the survival score in individuals with colorectal cancer with the AC/CC genotype in rs12373 was lower than the A.A. allele (rs12373A > C genotyping analysis detected that in disease-free survival (D.F.S.) with *p* value = 0.0008, and in overall survival (O.S.) with *p* value = 0.0001 in the dominant model) [21].

CD44, as the most putative marker of stem cells, might play a vital role in several cellular and molecular processes involved in migration and cell growth programs. In one study of 946 colorectal cancer patients and 989 controls, SNPs in 3′ UTR of CD44 genes revealed an association with increased susceptibility of colorectal cancer [2]. After genotyping based on allele-specific spectrometry and statistical analysis of three single-nucleotide variations rs11821102G/A, rs10836347C/T, rs13347C/T, it was found that C.T. and T.T. genotypes of rs13347, in comparison to the CC genotype, increased susceptibility of C.R.C. in the Han Chinese population. Moreover, C.R.C. patients with CT/TT genotypes showed an advanced C.R.C. with a 1.6-fold change in risk factors that increased the development of C.R.C. stages to stage III/IV (*p* value = 0.004). The alternation of C to the T nucleotide in rs13347 led to the removal of the miRNA hsa-miR-509-3p binding site. The increasing level of CD44 expression and transcription activity was illustrated based on the dual-Luciferase reporter assay. Furthermore, hsa-mir-509-3p mimics significantly decreased Luciferase activity in the rs2735383C allele in SW620 and SW116 cell lines (respectively *p* value = 0.007 and *p* value = 0.02). This result was confirmed by the transfection of C.R.C. cells with hsa-mir-509-3p mimic. Furthermore, carriers with the CT/TT genotype had a significant association with the rising risk of C.R.C. susceptibility. Moreover, patients with the T allele genotype in rs13347 (C.T. and T.T.) compared to the homozygote C allele expressed more amounts of CD44 mRNA. Therefore, it could be concluded that rs13347C/T in 3′ UTR of CD44 is associated with an increased risk and susceptibility of colorectal cancer, and the change in hsa-mir-509-3p binding in rs13347 position influenced the expression levels of CD44. 

**Figure 2:**
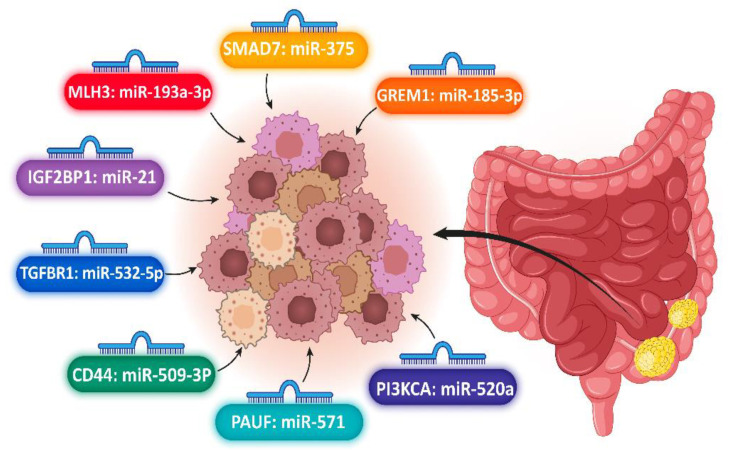
Differential expressions of pivotal miRNAs that targeted SNP single nucleotide polymorphism sites in candidate genes as biomarkers in prediction, early prognosis, diagnosis, and follow-up in colorectal cancers.

The risk of colorectal cancer incidence significantly increases in carriers with the C allele in SNP rs6504593 located in IGF2BP1 (Insulin-Like Growth Factor-2 Binding Protein-1) [22]. Based on the literature, the expression level of IGF2BP1 in different types of cancer is changed [23]. Xie and colleagues indicated that miR-21 bound to SNP rs6504593 in IGF2BP1 could regulate the expression of IGF2BP1 and develop colorectal cancer risk. In this study, they selected five SNPs, including rs6504593, rs6108, rs7337488, rs1049109, and rs7337488, of which rs1049109 and rs6504593 may increase progression of colorectal cancer. Furthermore, they found that rs6504593, which carried the TC/CC genotype, could be significantly elevated in non-drinkers and non-smokers. Notably, rs6504593 with the T.T. genotype increased in cases with a positive family history. Xie et al. stratified the samples based on older individuals, females, non-drinkers, non-smokers, with and without family history. Xie and colleagues demonstrated that rs1049109 CT/TT was predominantly enhanced in the subgroups. Hence, colorectal cancer could be considered as a multifactorial disorder in which environmental and genetic approaches could affect sporadic colorectal cancer etiology [24]. This case-control study indicated that among 1203 controls and 1147 cases, allele T in rs6504593 could dysregulate the IGF2BP1 as post-transcription via changing the binding affinity of miR-21 and mimic miR-21, which may stimulate cell proliferation and inhibit the apoptosis pathway. Gu and colleagues have found a correlation between colorectal cancer and the SNP rs1590 of transforming growth factor-b (TGFBR1), which acted as a promoter or tumor suppressor depending on the cellular context [25]. In addition, the rs1590 carrying GT/GG allele declined the risk of colorectal cancer compared with the T.T. allele. Based on this study, the risk of colorectal cancer in individuals with family history and older subjects was enhanced in comparison to the drinker and smoker cases. Furthermore, Gu et al. revealed that miR-532-5p may contribute to the SNP rs1590 in the 3’ UTR of TGFBR1. One study conducted a bioinformatic analysis involved in the DNA repair signaling pathway, and the selected genes included OGG1 rs1052133, MLH3 rs10862, ERCC1 rs735482, rs3212986, and rs2336219. Zhang et al. demonstrated that MLH3 rs108621 and ERCC1 rs3212986 are correlated with colorectal cancer risk [26]. Notably, for SNPs, rs3212986 AA genotype significantly increased the risk of cancer compared with the rs3212986 CC variant. In addition, the genotypes CC and TC in MLH3 rs108621 compared with TT allele and ERCC1 rs3212986 AA variant have predominantly enhanced the risk of colorectal cancer in males. The binding of miRNA-193a-3p to MLH3 rs108621 site was identified as a predictor factor of C.R.C. Hence, miR-193a-3p could act as a biomarker to predict colorectal cancer. Besides, the frequency of rs4939827 CC allele polymorphism of SMAD7 in the control was higher than in the C.R.C. cases [27]. In addition, in colorectal cancer patients, T-allele frequency was significantly increased compared with the control. In this study, there was a correlation between miR-375 and colorectal cancer, and the relative expression of miR-375 was significantly downregulated. As a result, rs4939827 SNP in SMAD7 and miR-375 might be considered as early diagnostic or prognostic biomarkers of colorectal cancer. Moreover, one study found that polymorphism of rs12915554 was located in the gremlin 1 (GREM1) gene and could elevate the GREM1 expression [10] that is a member of transforming growth factor-beta (TGF-β). Based on the bioinformatic analysis, the frequency of the C allele was more than the A allele. Therefore, rs12915554 potentially enhanced colorectal cancer through modulating the relative expression of the GREM1 gene via impairment of miR-185-3p binding site. Furthermore, Li and colleagues have indicated that hsa-miR-185-3p regulated the expression of GREM1 via binding to the miR-185-3p, which could promote the colorectal cancer risk. Moreover, the relative expression of these miRNAs was validated by Luciferase assay. Growing studies have revealed that miR-145 and miR-143 could target the KRAS gene in colorectal cancer [28]. Wang et al. found that the relative expression level of miR-145, miR-143, and KRAS genes significantly decreased in the cases of tissues compared with adjacent normal intestinal. In addition, the frequency of the C/T variant rs74693964 SNP was correlated with colorectal cancer. In this study, there was no association between KRAS rs1137196, rs712, miR-145 rs80026971, and miR-143 rs41291957 with the risk of colorectal cancer. A list of the miRNAs that bind to SNP sites in colorectal cancer are provided in Table 1. Moreover, we demonstrated vital miRNAs that could target SNP sites in candidate genes as biomarkers in colorectal cancer (Figure 2).

### 3.2. Gastric Cancer

Gastric cancer is identified as a malignancy with a multi-stage and multifactorial process. Moreover, based on the clinical evidence, *Helicobacter pylori* infection could have a critical role in stomach carcinogenesis via inducing inflammation [29]. In this process, epigenetics characteristics might alter the expression pattern of oncogenes and tumor suppressors and promote malignant or invasion through these conditions. IL-23/Th17 relation axis was identified as an inflammatory pathway involved in gastric carcinogenesis [30]. Inflammation pathway genes change expression under the influence of epigenetics and genetics factors such as SNPs, miRNAs, and miRNAs binding SNP sites. Four SNPs with the position ability to bind to the seed sequences of miRNAs, rs887796, rs1468488 of IL-17RA (Interleukin 17 Receptor A), rs10889677 of IL-23R (Interleukin 23 Receptor), and rs3748067 of IL-17A (Interleukin 17A) were genotyped with the restriction enzymes method, in 500 cancer-free subjects and 500 gastric cancer patients, in which gastric cancer was recently diagnosed by the histopathological methods [30]. Gene expression assay applied with real-time PCR, Western blot, and Luciferase were reported. In this study, Kaiyan Dong and collaborators found that T, CT, and C.T. + T.T. allele genotypes of rs3748067 adjusted for drinking status, smoking habits, and family history of gastric cancer are associated with a significant reduction in the gastric carcinogenesis risk. In regard to the rs10889677 genotyping test, the C allele and CC genotype are interrelated in increasing gastric cancer susceptibility. Furthermore, the Luciferase reporter assay showed that IL-17A gene expression was inhibited under the influence of miR-10a-3p. Relative gene expression of IL-17A was measured after efficient transfection of the miR-10a-3p inhibitor, negative control inhibitor, GenePharma-miR-10a-3p, and negative control vector to the SGC-7901 and BGC-823 cells lines. The RT-PCR results revealed that gene expression of IL-17A in GenePharma-miR-10a-3p compared to the negative control vector group was lower in the SGC-7901 cell line.

Moreover, the relative expression of IL-17A in the miR-10a-3p inhibitor group was higher than the negative control inhibitor. Besides, relative expression of miR-10a-3p in contrast with internal miRNA U6 in four transfected groups was assessed and indicated a significantly higher expression in GenePharma-miR-10a-3p and was downregulated in miR-10a-3p inhibitor group in comparison to their negative control groups. Moreover, Western blot assay of IL-17A and internal protein GAPDH from four transfection groups in SGC-7901 and BGC-823 cells lines indicated a significant lower expression of IL-17A in the GenePharma-miR-10a-3p group compared to the negative control vector, while the miR-10a-3p inhibitor-transfected group showed high overexpression of the IL-17A protein in comparison to the NC inhibitor.

In one bioinformatics/experimental analysis, the effect of SNPs on the binding affinity of MTMR3 gene position on miRNA-181a and regulations of post-transcriptional gene expression were evaluated via microarray data, reverse-transcription PCR (RT-PCR), SNPs genotyping, and Luciferase reporter assay [31]. Results of rs12537 genotyping in 500 gastric cancer patients and 502 control subjects confirmed the binding to miR-181a [31]. It was shown that the C.T. and T.T. alleles were associated with significantly high gastric cancer risk (*p* = 0.029). Moreover, heterozygous genotype carriers (C.T.) in comparison to the homozygous genotype (CC) in rs12537 had a low level of mRNA expression of the MTMR3 gene; however, the expression of miR-181a was not significantly differentiated. Verification of the miR-181a effect on MTMR3 genes by Luciferase reporter assay illustrated that the enhancement of miR-181a expression had major suppressive influences in rs12537C > T-substituted allele carriers.

Tyrosine-protein kinase erbB-3 receptor, one member of the epidermal growth factor receptor family encoded by the ERBB3 gene in humans, is involved in the proliferation and metastatic processes via several molecular and cellular signaling pathways. Previous studies have indicated that this oncogene pattern with an overexpression gene profile in gastric cancer cases was strongly associated with cancer susceptibility. Yaxiang Shi et al. in 2017 showed that rs3202538 (G.T. and T.T. genotype) in the 3’ UTR region of ErbB3 was related to gastric cancer development risk through removing the targeting site of miRNA-204 and miRNA-211 in the rs3202538 location [32]. Furthermore, the rs3202538 genotyping analysis and clinical–pathological features survey of 851 gastric cancer patients compared to 799 normal subjects demonstrated that carriers of T genotype were presented with significantly large tumor size and poor differentiation, with high potential of metastasis. The results of real-time PCR confirmed such differences in ErbB3 transcription in rs3202538 genotypes. Finally, this study indicated that the G/T SNP might serve as a tumor promoter and poor prognosis indicator in gastric cancer by affecting the binding of miR-204 and miR-211 on the 3’ UTR of ErbB3. Furthermore, the outputs of this study indicated that the SNP in 3’ UTR of ERbB3 could affect the binding affinity of both miR-204 and miR-211 in the T mutant allele compared to the wild-type genotype (G.G.). Hence, the T mutant allele affected the function of post-transcriptional regulation, leading to abnormal expression levels of ErbB3. In addition, post-transcriptional regulation by miR-204 and miR-211 might be attenuated dramatically due to the G > T SNP in 3’ UTR of ErbB3. A five-year survival rates study, which was performed on 851 gastric cancer cases, showed that 344 patients had follow-up data, and 36 patients had recurrent gastric cancer. Furthermore, the overall 5-year survival score analysis indicated that TT/GT genotype groups with the lowest rate (15.72%) among others had significant differences in survival rate. 

IRF-1 genes (Interferon (I.F.N.) regulatory factor-1) are the first specified subtype of the I.R.F. family that have physiological roles in tumor prevention, immune development, and establishing host defense against pathogens. The result of one study on the effects of the IRF-1 gene as a tumor-inducer factor in gastric cancer susceptibility in 819 gastric cancer case patients and 765 control had shown that C/G genotype in rs56288038 of IRF-1 gene was associated with the occurrence of gastric cancer conditions (*p* value = 0.0001) [33]. In this research, Wang et al. confirmed that SNP rs56288038 with C/G genotype was targeted by miR-502-5p and could increase the affinity of miR-502-5p to the SNP binding site of IRF-1. Therefore, the overexpression of miR-502-5p and increasing the binding affinity of miRNA to polymorphism sites influenced the expression of theIRF-1 gene. Luciferase assay and immunohistochemistry stain revealed that SNP in the 3’ UTR of IRF-1 might affect the binding affinity of miR-502-5p, resulting in abnormal expression of IRF-1 levels. Furthermore, C.G. and G.G. genotypes of rs56288038 in the 3’ UTR of IRF-1 were significantly associated with gastric cancer risk. Moreover, the clinical features showed that C.G. and G.G. genotypes are significantly associated with H. pylori infection (*p* value = 0.0001), poor differential grade (*p* value = 0.0001), tumor size (*p* value = 0.0001), positive metastatic condition (*p* value = 0.0001), and depth of invasion (*p* value = 0.0001). The results of 5-year survival rate indicated that C.G. and G.G. genotype groups had significantly lower survival scores and worse rates of prognosis in comparison to the CC genotype group.

In another case-control research, 500 gastric cancer patients and 500 matched healthy subjects from the China population were considered in which SNPs in 3’ UTR of interleukin 1 (IL-1) family genes were identified as vital points in the susceptibility and development of gastric cancer [29]. A bioinformatics survey in SNP and miRNA databases targeted SNPs in 3’ UTR regions of IL-1 family genes by miRNAs. Genotyping of SNPs, rs9005, rs2472188, rs2515401, rs2856836, rs3732131, rs1135354, rs3771157, rs957201, rs3180235, and rs2515402 were conducted by RFLP-PCR and allele-specific PCR (AS-PCR) methods. Among 10 SNPs genotypes, variant genotypes of rs9005 in 3’ UTR regions of Interleukin 1 Receptor Antagonist (IL-1RN) consisting of G.A. heterozygous genotype and A.A. homozygous genotype were associated with a high risk of gastric cancer. Allelic frequencies of two polymorphisms (rs2472188 and rs2515401) in 3’ UTR of member 5 of the Interleukin 1 gene family (IL-1F5), were investigated, and genotyping outcomes showed that the G.C. genotypes of rs2472188 compared to G.G. allele were significantly related with gastric cancer risk. Carriers of the C.T. genotype compared to the wild genotype CC in rs2515401 were revealed to have a high risk of gastric cancer. According to the miRNA prediction databases, miR-197 targeted the 3’ UTR region of IL1-F5. Moreover, real-time PCR, Western blot, and Luciferase methods were performed for the investigation of influencing of miR-197 on IL1-F5 and measurement of their expression. The relative expression of I.L. 1-F5 after transfection of miR-197 in two human colorectal cancer cell lines, SGC-7901 and BGC-823, was diminished, and miR-197-inhibitor had reversed the gene expression pattern of IL1-F5. Hence, they concluded that miR-197 could negatively regulate IL1-F5 expression. Based on the obtained results, Xiaolin Chen et al. suggested that IL1-F5 as a potential target gene for miR-197 could be engaged in gastric cancer development.

**Figure 3:**
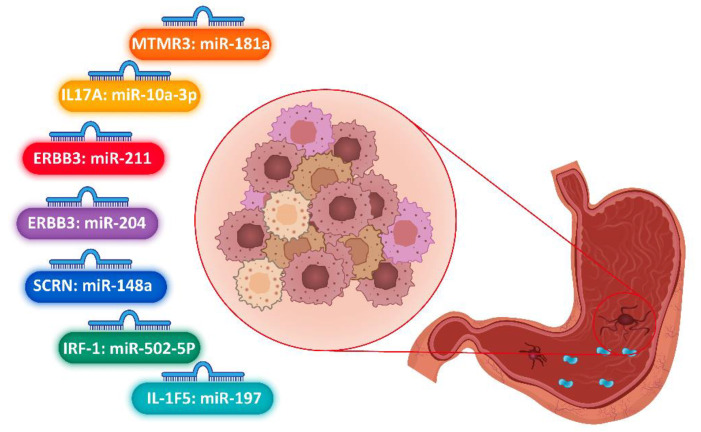
Differential expressions of pivotal miRNAs that targeted SNP sites in candidate genes as biomarkers in prediction, early prognosis, diagnosis, and follow-up in gastric cancers.

Furthermore, in another study that was performed between 2006 to 2010 among the gastric carcinoma China population, 753 gastric carcinoma patients and 949 cancer-free subjects were evaluated based on pathohistological surveys of neoplasm tissue, quantitative gene expressions, and genotyping. According to the in silico study for miRNA-148a-related to SNP sites, two polymorphisms were detected: Prodynorphin (PDYN) rs2235749 and Secernin-1 (SCRN1) rs6976789 [34]. Genotyping analysis of these SNPs in the case and control individuals manifested that the variation genotype C > T in rs6976789 of SCRN1 gene had a significant association with high gastric cancer risk. Furthermore, the cumulative survival score of gastric carcinoma carriers with T.T. genotypes of SCRN1 rs6976789 was lower than CC and C.T. genotypes. The binding ability of miR-148a in rs6976789 polymorphism site in 3’ UTR of SCRN1 gene was confirmed by Luciferase reporter assay. Relative expression of the SCRN1 gene and miR-148a between tumor and normal tissues indicated that this gene in the tumor sample was overexpressed (*p* < 0.001), and miRNA-148a was decreased in the tumor tissue compared to normal tissue (*p* < 0.001). Moreover, the expression of the SCRN1 gene in carriers with the T.T. genotype was greater than the CC and C.T. genotypes. The overall outcomes of this research manifested a pivotal role of SCRN1 and its single nucleotide polymorphisms in susceptibility, prognosis, and development of gastric carcinoma. We summarized these studies related to gastric cancer in Table 2. Furthermore, we designed essential miRNAs that might bind to SNP sites in candidate genes as biomarkers in gastric cancer (Figure 3).

### 3.3. Assessment of the Risk of Bias and Quality

The risk of bias was evaluated with the Newcastle–Ottawa Scale checklist. We found fifteen studies with a low risk of bias (three with 9/9, four with 8/9, and seven with 7/9 scores). Moreover, only one article indicated a moderate risk of bias with a score of 6/9. In most studies, the calculation and estimation of sample size were not considered, and there is no explanation of the response characteristics. In addition, in one study, the selection of controls was not detected. Notably, most of the studies did not identify the stage of cancer separately. All evidence received all exposure criteria, such as appropriate ascertainment of exposure and case controls (Table 3). In addition, we assessed the quality of the study based on the four eligibility criteria, including evidence of bias, evaluation of outcome, appropriateness and reporting of exclusion and inclusion as previously reported, and validation of outcome. In this systematic analysis, we found that one study was considered low quality; whereas in most studies, the quality was high (Table 3).

## 4. Discussion

This systematic assessment aimed to investigate the interaction between miRNA–mRNA in SNP sites in gastrointestinal cancer susceptibility. The miRNAs with the ability of post-transcriptional regulation of gene expression might be affected by various conditions such as molecular and biological elements, genetics, epigenetics factors, physiological and environmental conditions [3,4,5,6]. Conversely, the SNP available in the targeted sequences could be influenced by miRNA–mRNA binding affinity [18,19]. Moreover, different mechanisms might have positive or negative consequences for these regulating patterns. In this systematic review, among 64,006 articles obtained from PubMed, Web of Science, Embase, and Scopus databases, 15 articles were selected, which were about miRNA binding to polymorphism sites in gastrointestinal cancers. We engaged SNPs presented in 3’ UTR sequences of pivotal genes with the miRNA binding ability to SNP sites. Moreover, we marked pivotal single nucleotide variation in essential genes involved in gastrointestinal carcinogenesis. We also stratified remarkable SNPs as molecular–biological markers in the diagnosis and prognosis of gastrointestinal carcinomas such as PIK3CA rs141178472, PAUF rs12373, CD44 rs13347, rs6504593, and rs1049109 in 3’ UTR of IGF2BP1, TGFBR1 rs1590, MLH3 rs108621, ERCC1 rs3212986, SMAD7 rs4939827, GREM1 rs12915554, KRAS rs74693964, IL-23R rs10889677, IL-17A rs3748067, MTMR3 rs12537, ERBB3 rs3202538, IRF-1 rs56288038, IL-1RN rs9005, IL-1F5 rs2472188 and rs2515401, SCRN1 rs6976789. In our study, the binding of miRNAs to SNP sites was especially confirmed by technical methods. Hence, we found differential expressions of pivotal miRNAs that targeted SNP sites. The miR-520a: PIK3CA, miR-571: PAUF, miR-509-3p:CD44, miR-21: IGF2BP1, miR532-5p: TGFBR1, miR-193a-3p: MLH3, miR-375: SMAD7, miR-185-3p: GREM1, miR-145 and miR-143: KRAS, miR-10a-3p:IL-17A, miR-181a: MTMR3, miR-204 and miR-211: ERBB3, miR-502-5p: IRF-1, miR-197:IL-1F5, miR-148a: SCRN1 were identified as strong post-transcriptional regulators. PAUF rs12373: miR-571, MLH3 rs108621: miR-193a-3p, SMAD7 rs4939827: miR-375 were detected as biomarkers in the prediction, early prognosis, diagnosis, and follow-up in gastrointestinal cancers.

Qi-Xian Wang et al., in their systematic and meta-analysis review, reported that CA199, C.E.A., and CA724 were available as the non-specific biomarkers for diagnosis of a wide variety of neoplasms [35], such that necessity needed novel biomarkers for early diagnosis of gastrointestinal carcinomas. In previous systematic research, studies were included with bioinformatics prediction and weak confirmation, while we determined the minimal criteria for inclusion research. Gholami and colleagues characterized SNPs in miRNA binding sites in colorectal cancer risk, while we engaged these biological markers in various gastrointestinal carcinoma [36]. They performed a merged systematic review and meta-analysis that included 85 research studies obtained from two previous systematic reviews about miRs-binding to SNP sites in colorectal carcinoma susceptibility. In other studies, Carter and colleagues found that 31 miRNAs regulate colorectal cancer. They also indicated that miRNAs with high specificity and sensitivity could be a suitable and invasive candidate for detecting colorectal cancer. Although Carter et al. found that miRNA might regulate C.R.C., they did not detect the association between SNPs and miRNAs [37]. We found that 20 SNPs and 17 miRNAs were involved in C.R.C. and G.C. according to our strict criteria that are shown in Table 4.

Wang et al. indicated that the relative expression of 47 miRNAs was dysregulated in gastric cancer [35]. They employed published papers only from the PubMed online database, and they did not consider the role of SNP binding to miRNAs in gastric cancer.

Nevertheless, our comprehensive systematic review possesses certain limitations. The disadvantage of these retrieved research studies is that consideration was not specified for gastric and colorectal cancer risk agents, such as infections, diet, genetics, lifestyles, and other risk elements that might participate in differential expression of miRNA profiles. Moreover, our systematic review did not engage bioinformatics data, microarray analysis outcomes, cell culture studies, or animal research. Hence, applying these exclusion criteria might be a marked limitation in results. Conversely, in this systematic research, we ignored some regions of the digestive system for surveys such as mouth, esophagus, duodenum, and we only employed stomach and colorectal cancers as common cancers.

## 5. Conclusions

This study revealed the role of SNP and binding miRNAs in gastrointestinal cancers that might be a principal and essential progression, invasion, and susceptibility of cancers. In addition, based on this systematic review, we found that the profile of SNP and miRNAs might be a convenient and candidate approach to the prognosis and diagnosis of gastric and colorectal cancers. Furthermore, the predictive power in combination biomarkers is considered to be a robust approach for early diagnosis.

## Figures and Tables

**Table 1:** Characteristics of studies that included the feature of patients and miRNA binding to SNP sites in colorectal cancer.

	Population		Region	Variables			Age		Gender Control		Gender Patient		Stage		
ID	Patient	Control	Country	miRNAs	SNP	Gene	Patient	Control	Male	Female	Male	Female	Low	intermediate	high
Xie, 2018	1147	1203	China	miR-21	rs6504593	IGF2BP1	60.0 ± 12.6	59.9 ± 14.3	698	505	702	445	85	880	182
Gu, 2018	1147	1203	China	miR-532-5p	rs1590	TGFBR1	60.0 ± 12.6	59.9 ± 14.3	698	505	702	445	85	880	182
Zhang, 2017	200	200	China	miR-193a-3p	rs10862, rs3212986	MLH3, ERCC1	62.18 ± 12.637	61.59 ± 13.153	89	89	111	111	ND	ND	ND
Shaker, 2016	86	36	Egypt	mir-375	rs4939827	SMAD-7	50.4 ± 12.4	46.8 ± 8.7	19	17	59	19	ND	ND	ND
Li, 2017	1841	1837	China	hsa-miR-185-3p	rs12915554	GREM1	40 ± 24.8	40 ± 26.1	1026	811	1025	816	1841	ND	ND
Wang, 2021	507	497	China	miR-143, miR-145	rs74693964	KRAS	62.55 ± 11.88	62.75 ±11.99	288	207	329	178	ND	ND	ND
Ding, 2015	386	394	China	miR-520a	rs141178472	PIK3CA	60.1 ± 12.3	60.7 ± 12.9	229	165	216	170	38	308	40
Kim, 2015	831	-	South Korean	miR-571	rs12373, rs3757417T	PAUF	63 ± 20.3	ND	ND	ND	55.3 ± 2.4	43.2 ± 1.3	150	665	16
Wu, 2015	946	989	China	hsa-mir-509-3p	rs13347, rs10836347 rs11821102G/A	CD44	55 ± 24.6	58 ± 22.7	535	454	519	427	84	606	256

**Table 2:** Characteristics of studies that included the feature of patients and miRNAs binding to SNP sites in Gastric cancer.

	Population		Region	Variables			Age		Gender Control		Gender Patient		Stage		
ID	Patient	Control	Country	miRNAs	SNP	Gene	Patient	Control	Male	Female	Male	Female	Low	intermediate	high
Dong, 2017	500	500	China	hsa-miR-10a-3p	rs3748067	IL17A	57.93 ± 11.88	57.27 ± 12.15	124	376	124	376	ND	ND	ND
Lin, 2012	500	502	China	miR-181a	rs12537	MTMR3	60 ± 13	60 ± 15	319	183	314	186	ND	ND	ND
Shi, 2017	851	799	China	miR-204 miR-211	rs3202538	ErbB3	≤50	>50	319	480	318	533	214	294	343
Wang, 2016	819	765	China	MiR-502-5p	Rs56288038	IRF-1	≤50	>50	319	501	318	480	214	294	311
Chen, 2015	500	500	China	miR-197	rs2472188	IL1-F5	58.03 ± 11.89	57.24 ± 12.15	376	376	124	124	ND	ND	ND
Song, 2014	753	949	China	miR-148a	rs6976789, rs2235749	SCRN1	65 ± 19	64 ± 18	628	321	512	241	222	431	12

**Table 3:** Evaluated risk of bias and quality assessment of included evidence in colorectal and gastric cancer.

Study ID	Selection				Comparability		Exposure			Score	Quality Assessment
Xie, 2018	1	1	1	1	1	0	1	1	1	8	5
Gu, 2018	1	1	1	1	1	1	1	1	1	9	5
Zhang, 2017	1	0	1	1	1	0	1	1	1	7	4
Shaker, 2016	1	1	1	1	1	1	1	1	1	9	5
Li, 2017	1	0	1	1	1	0	1	1	1	7	4
Wang, 2021	1	0	1	1	1	0	1	1	1	7	4
Wu, 2015	1	1	1	1	1	1	1	1	1	9	5
Kim, 2015	1	1	0	1	0	0	1	1	1	6	3
Ding, 2015	1	1	1	1	1	0	1	1	1	8	5
Shi, 2017	1	0	1	1	1	1	1	1	1	8	5
Dong, 2017	1	0	1	1	0	1	1	1	1	7	4
Wang, 2016	1	0	1	1	0	1	1	1	1	7	4
Chen, 2015	1	1	1	1	0	0	1	1	1	7	4
Peng Song, 2014	1		1	1	1	1	1	1	1	8	5
Yong Lin, 2012	1	1	1	1	0	0	1	1	1	7	4

**Table 4:** The summary of expression pattern alteration miRNAs in binding to SNP sites is based on the inclusion criteria as biomarkers in prediction, early prognosis, diagnosis, and follow-up in gastrointestinal cancers.

Study ID	Genes	SNPs	miRNAs	Expression miRNAs Pattern in Colorectal Cancer	Expression miRNAs Pattern in Gastric Cancer	Biomarker Categories	Significant Scores
Ding, 2015	PIK3CA	rs141178472-T allele	miR-520a	↑	ND	Prognosis	95% CI: 1.716 (1.084–2.716), *p* = 0.022
Kim, 2015	PAUF	rs12373-A allele	miR-571	↑	ND	Prognosis	95% CI: 1.59 (1.21–2.08), *p* = 0.0008
Wu, 2015	CD44	rs13347-CT and TT alleles	miR-509-3p	↓	ND	Prognosis	95% CI: 1.79 (1.50–2.17), *p* = 0.004
Xie, 2018	IGF2BP1	rs1049109 T allele and rs6504593	miR-21	↓	ND	Diagnosis	95% CI: 1.23 (1.07–1.41), *p* = 0.004 and 95% CI: 1.19 (1.04–1.36), *p* = 0.011
Gu, 2018	TGFBR1	rs1590 GT and GG alleles	miR532-5p	↓	ND	Prognosis	95% CI: 0.82 (0.68–0.97) *p* = 0.024
Zhang, 2017	MLH3	rs108621 CC and TC alleles	miR-193a-3p	↑	ND	Prognosis	95% CI: 6.237 (1.298–29.966), *p* = 0.013 And 95% CI: 2.079 (1.171–3.691), *p* = 0.014
Zhang, 2017	ERCC1	rs3212986 AA alleles	-	ND	ND	Prognosis	95% CI: 4.043 (1.261–12.968), *p* = 0.021
Shaker, 2016	SMAD7	rs4939827 T allele	miR-375	↓	ND	Diagnosis	*p* = 0.01
Li, 2017	GREM1	rs12915554 C allele	miR-185-3p	↓	ND	Prognosis	95%CI: 1.43 (1.04–1.95), *p* = 0.026
Wang, 2021	KRAS	rs74693964 C and T alleles	miR-145	↓	ND	Prognosis	95% CI: 1.901 (0.943–3.835), *p* = 0.001
Dong, 2017	IL23R	rs10889677 CC	-	ND	-	Diagnosis	95% CI: 2.22 (1.27–3.87), *p* = 0.01
	IL17A	rs3748067 T, CT and CT + TT alleles	miR-10a-3p	ND	↑	Diagnosis	95% CI: 0.58 (0.43, 0.77), *p* = 0.01
Lin, 2012	MTMR3	rs12537 CT and TT alleles	miR-181a	ND	↑	Prognosis	95% CI: 1.72 (1.36–2.16), *p* = 3.99 × 10^−5^
Shi, 2017	ERBB3	rs3202538 GT and TT alleles	miR-204	ND	↓	Diagnosis	95% CI: 1.89 (1.48–2.01), *p* = 0.0001
Shi, 2017	ERBB3	rs3202538 GT and TT alleles	miR-211	ND	↓	Diagnosis	95% CI: 4.32 (1.34–1.88), *p* = 0.0001
Wang, 2016	IRF-1	rs56288038 C and G alleles	miR-502-5p	ND	↑	Diagnosis	95% CI: 3.96 (1.52– 2.11), *p* = 0.0001
Chen, 2015	IL-1F5	rs2472188 GC and GC + CC alleles	miR-197	ND	↑	Prognosis	95% CI: 1.51 (1.15,1.99), *p* = 0.01 And 1.37 (1.06,1.77), *p* = 0.01
Chen, 2015	IL-1F5	rs2515401 C.T. alleles	miR-197	ND	↑	Prognosis	95% CI: 1.36 (1.04,1.76), *p* = 0.01
Song, 2014	SCRN1	rs6976789 allele	miR-148a	ND	↓	Prognosis	95% CI: 2.47, (1.21–5.05), *p* = 0.009
